# The role of social anxiety in suicidal risk among university students in Northern Morocco

**DOI:** 10.1192/bji.2025.10055

**Published:** 2026-02

**Authors:** Sara Echater, Fadila Bousgheiri, Karima Sammoud, Yassine Benhaddouch, Saloua Lemrabett, Meftaha Senhaji, Adil Najdi, Adil El Ammouri

**Affiliations:** 1 Assistant Professor of Psychiatry, Department of Psychiatry, Faculty of Medicine and Pharmacy of Tangier, Mohammed VI University Hospital, Abdelmalek Essaâdi University (UAE), Tangier, Morocco. Email: dr.sara919@gmail.com; 2 Assistant Professor of Community Medicine, Department of Epidemiology, Public Health, and Social Sciences, Faculty of Medicine and Pharmacy of Tangier, Abdelmalek Essaâdi University (UAE), Tangier, Morocco; 3 Resident Doctor of Community Medicine, Department of Epidemiology, Public Health, and Social Sciences, Faculty of Medicine and Pharmacy of Tangier, Abdelmalek Essaâdi University (UAE), Tangier, Morocco; 4 Professor of Neuroscience, Department of Biology and Health, Faculty of Sciences, Abdelmalek Essaâdi University (UAE), Tetouan, Morocco; 5 Head of Department and Full Professor of Community Medicine, Department of Epidemiology, Public Health, and Social Sciences, Faculty of Medicine and Pharmacy of Tangier, Abdelmalek Essaâdi University (UAE), Tangier, Morocco; 6 Head of Department and Associate Professor of Psychiatry, Department of Psychiatry, Faculty of Medicine and Pharmacy of Tangier, Mohammed VI University Hospital, Abdelmalek Essaâdi University (UAE), Tangier, Morocco

**Keywords:** Social anxiety disorder (SAD), suicidal risk, university students, depression, Morocco

## Abstract

**Background:**

Psychiatric disorders are a major risk factor for suicidal behaviors. However, increasing attention is being given to anxiety disorders, which have also been associated with suicidal risk.

**Aims:**

This study aims to examine the prevalence of social anxiety disorder (SAD) among university students, explore its association with suicidal risk and assess the role of depression as a potential confounding factor in this relationship.

**Method:**

We conducted a cross-sectional, multicentre study involving students from Abdelmalek Essaâdi University. Data were collected face-to-face using a structured questionnaire designed on the REDCap platform. The Moroccan Arabic version of the MINI (Mini International Neuropsychiatric Interview) was used to assess SAD, depression and suicidal risk. All students present and consenting were included. Data were analysed using descriptive statistics and multivariate logistic regression to evaluate the independent association between SAD and suicidal risk.

**Results:**

Among the 1168 students surveyed, 59.1% were women, and the average age was 20.63 years. The prevalence of social anxiety was 9.9% (95% CI: 8.3–11.8). Social anxiety disorder is an independent risk factor for suicide, even after adjustment for other well-known variables such as depression, with an adjusted odds ratio of 1.84 (95% CI: 1.12–3.04).

**Conclusion:**

SAD is a major risk factor for suicidal behaviors. These results highlight the importance of early identification and appropriate management of SAD among students in order to prevent suicidal risks.

Social anxiety disorder (SAD) is an anxiety disorder characterised by a marked and persistent fear of social or performance situations, where the individual fears being judged, humiliated or rejected. This excessive anxiety can lead to significant distress and impairment in daily functioning, often resulting in avoidance of social interactions.^1^

SAD typically begins in adolescence, with a lifetime prevalence estimated at 12.1%, making it one of the most common psychiatric disorders.^2^ Its prevalence has been particularly studied among university students. A meta-analysis conducted in Ethiopia reported a prevalence of 28.05% in this group,^3^ while studies conducted among university students in Sweden and Australia found rates of 16.1^4^ and 30%,^5^ respectively.

Psychiatric disorders are a major risk factor for suicidal behaviors. According to a meta-analysis of 27 studies involving 3275 suicide cases, 87.3% (standard deviation of 10.0%) of individuals had received a mental health diagnosis prior to their death,^6^ with mood disorders and personality disorders being the most frequently involved.^7^ However, there is growing interest in anxiety disorders, which have also been linked to suicide risk.^8^ A review and meta-analysis involving 65 studies with adults and adolescents, demonstrated a significant association between anxiety disorders and suicidal thoughts (odds ratio 1.49; 95% CI: 1.18–1.88), as well as suicide attempts (odds ratio 1.64; 95% CI: 1.47–1.83).^9^

Among anxiety disorders, SAD may play a particularly significant role in suicidality. In fact, Bentley et al identified it as a risk factor for suicidal thoughts and attempts. A more recent meta-analysis confirmed this association by showing a significant correlation between social anxiety and suicide risk in young people aged 10 to 25 years.^10^ Several mechanisms could explain this association, including social isolation, unfavourable social comparisons and difficulty seeking psychological support.^11^

Social anxiety is often associated with depression, with more than 20% of individuals with SAD experiencing a major depressive episode.^12,13^ Therefore, it is possible that the observed association between SAD and suicidality is partly explained by the concurrent presence of depressive symptoms, which is poorly understood and discussed in the literature.

In this context, our study aims to examine the prevalence of SAD among university students and explore its association with lifetime suicide attempts, current suicidal ideation and suicide risk. We will also assess the role of depression as a potential confounding factor in this relationship.

## Method

### Study design

This is a cross-sectional, multicentre, descriptive and analytical study conducted in 2023 among university students at Abdelmalek Essaâdi University (UAE), located in the northern region of Morocco. The study encompassed all university institutions affiliated with UAE within the cities of Tangier, Tetouan, Larache and Al Hoceima.

### Population and sampling

A stratified random sampling method, based on institution and gender, was employed to obtain a representative sample of the student population at UAE. The sample size was determined using the following parameters: an expected prevalence of *p* = 50%, a significance level (*α*) of 0.05, a 95% CI and a precision of 3%. Based on these criteria, the minimum required sample size was calculated to be 1067 students. This final sample was proportionally distributed according to the total student population of each institution and gender, using official data provided by UAE for its different sites.

The study focused on enrolled university students aged over 18 years. Participation was voluntary and anonymous. All students aged 18 and above who agreed to participate were included, while students under 18, as well as those who refused to participate, were excluded from the study.

### Data collection tools

Data were collected using a form designed with the REDCap software, a secure platform widely used in clinical and epidemiological research.^14,15^ REDCap was created in 2004 at Vanderbilt University (Nashville, TN, USA) and is accessible at https://projectredcap.org. The Mohammed VI University Hospital in Tangier has institutional access to this platform.

The survey began in March 2023 and spanned one year, avoiding exam periods. The investigators were healthcare professionals, including physicians, mental health nurses and medical interns, all of whom were trained and familiar with the study protocol and the questionnaire used. Participants were recruited through random sampling in various locations frequented by students, including libraries, cafeterias and lecture halls. Each student was informed about the purpose of the study before providing their informed consent.

The survey was conducted face-to-face using a structured questionnaire consisting of two sections.

#### Sociodemographic data and history

Age, gender, marital status, residence before university studies (urban/rural), current city of residence, residential status (living alone, sharing with friends or living with family), level of education, history of psychoactive substance use and history of psychiatric follow-up.

#### Clinical evaluation

SAD, depression and suicidal risk were assessed using the Mini International Neuropsychiatric Interview (MINI), in its Moroccan Arabic dialect version validated by the University Psychiatric Center Ibn Rushd.^16^ The MINI is a brief structured interview designed to diagnose the main psychiatric disorders from Axis I of the DSM-IV and ICD-10.

### Social anxiety disorder evaluation

The MINI assesses social anxiety disorder using four questions that explore intense and persistent fear of social or performance situations, disproportionate fear in relation to the actual situation, avoidance behaviours and the impact of this fear on daily functioning. It specifically focuses on current (past month) symptoms of social anxiety, and the diagnosis is considered if these symptoms have been present during this period.

### Depression evaluation

The MINI explores the presence of a major depressive episode through questions targeting the duration and intensity of depressive mood, anhedonia, sleep disturbances, weight changes, fatigue, decreased concentration, excessive guilt and suicidal thoughts. A depressive episode is identified if a sufficient number of DSM-IV criteria are met.

### Suicidal risk evaluation

The MINI includes six questions assessing suicidal risk: wish to be dead, desire to harm oneself, suicidal thoughts, suicidal planning, suicide attempt in the last month and lifetime suicide attempt. A ‘yes’ answer to any of these questions indicates the presence of current suicidal risk. This risk is then classified into three levels (low, moderate and/or high) based on the number of positive answers and the nature of the responses (presence of a plan or recent attempt, etc.).

### Statistical analysis

Statistical analysis was performed using IBM SPSS Statistics version 26 (IBM, Armonk, NY, USA), running on macOS. The software is available at https://www.ibm.com/products/spss-statistics. Descriptive analysis presented quantitative variables as means and s.d.s, and qualitative variables as percentages. A univariate analysis was conducted using the *χ* ^2^ test to compare percentages and the Student’s *t*-test to compare group means. Finally, a logistic regression model was applied to examine the independent and interactive associations between depression, social anxiety and suicidal risk.

### Ethical considerations

The questionnaire was administered after obtaining verbal informed consent from the students. Verbal consent was witnessed and formally recorded. The collected data were stored within the research laboratory of the faculty, with access limited to the research team members.

The study received approval from the Hospital-University Ethics Committee of Tangier (CEHUT) under the number 15/2023.

## Results

### Descriptive analysis

Among the 1168 students surveyed, 59.1% (690) were female and 40.9% (478) were male, with a sex ratio of 0.69. The average age was 20.63 years (s.d. = 2.9), and 94.9% were single. Regarding their place of residence before university, 10.8% (124) of students came from rural areas and had to move to continue their studies, while 89.2% (1025) were already living in urban areas. Currently, 23.2% (271) live alone or share accommodation with friends, while 76.8% (896) live with their family.

In terms of academic level, 56.6% (659) were in their first or second year of university, while 43.2% (505) were pursuing a bachelor’s degree or beyond.

Regarding personal history, 12.6% (144) reported using one or more psychoactive substances (alcohol, tobacco, cannabis, etc.), and only 5.3% (62) self-reported being followed for a psychiatric disorder, Among these, 38% reported anxiety disorders (including generalised anxiety disorder, panic disorder and social anxiety disorder), 32% depressive disorders, 10% histrionic personality disorder, and smaller proportions reported bipolar disorder (4%), schizophrenia (4%) obsessive–compulsive disorder (2%) and sleep disorders (2%).

The prevalence of social anxiety disorder was estimated at 9.9% (115) (95% CI: 8.3–11.8).

### Bivariate analysis

Social anxiety disorder and suicidal risk were significantly higher among females, students aged 20 or younger and those with a current major depressive episode or a history of psychiatric disorder (Table 1).

**Table 1:** Bivariate analysis of demographic and clinical factors related to social anxiety disorder (SAD) and suicidal risk

Variable	SAD		Suicidal risk	
	(%)	*p*-value	(%)	*p*-value
**Gender**		**0.003**		**0.000**
Male	6.8		7.1	
Female	12.1		14.1	
**Age**		**0.017**		**0.015**
≤20 years	12.4		14	
>20 years	8.2		9.4	
**Origin**		0.772		0.070
Rural	10.6		6.5	
Urban	9.7		11.9	
**Residential status**		0.951		0.234
Alone	10.0		9.2	
Living with family	9.9		11.8	
**Current major depressive episode**		**<0.001**		**0.000**
No	6.9		3.9	
Yes	19.4		34.2	
**Psychoactive substance use**		**0.038**		0.199
No	10.8		10.9	
Yes	5.3		14.6	
**History of psychiatric disorder**				
No	10.5	**0.001**	10.1	
Yes	21		32.3	**0.000**

Bold values indicate statistical significance with *p* < 0.05.

The results show a higher prevalence of suicidal thoughts, suicide attempts and an increased suicidal risk in individuals with social anxiety disorder, with a statistically significant association (Table 2).

**Table 2:** Bivariate analysis of the association between social anxiety disorder (SAD) and suicidal risk

Variable	SAD		*p*-value
	Yes (%)	No (%)	
Suicidal ideation	27.9	6.9	**<0.001**
Suicide attempt in the past month	5.5	0.9	**<0.001**
Lifetime suicide attempt	11.6	4.6	**0.002**
Suicide risk	28.7	9.4	**<0.001**

Bold values indicate statistical significance with *p* < 0.05.

### Multivariate analysis

We introduced the variables of interest into the initial model with the goal of controlling for confounding variables. The variables included in the initial model were gender, age, social anxiety disorder, current major depressive episode, psychoactive substance use and psychiatric disorder history. We kept the initial model, without applying stepwise regression. The binary logistic regression identified five variables significantly associated with suicidal risk, as shown in Table 3.

**Table 3:** Relationship between suicidal risk and social anxiety disorder (SAD), after adjusting for confounding factors using binary logistic regression method

Variables	Adjusted odds ratio	95% CI	*p*-value
Gender (female)	1.844	1.118–3.043	**0.017**
Age	0.780	0.509–1.196	0.225
SAD	2.431	1.448–4.081	**0.001**
Current major depressive episode	10.834	6.948–16.894	**0.000**
Psychoactive substance use	2.154	1.144–4.058	**0.018**
History of psychiatric disorder	3.093	1.560–6.132	**0.001**

Bold values indicate statistical significance with *p* < 0.05.

Among these variables, the presence of social phobia was identified as a suicidal risk factor, with an adjusted odds ratio of 1.844 (95% CI: 1.118–3.043). This means that after adjusting for confounding variables such as age, current major depressive episode, psychiatric disorder history and psychoactive substance use, individuals with social anxiety disorder have nearly double the suicidal risk compared with those without this condition.

Adjusting for these confounders allows us to isolate the specific effect of social anxiety disorder while accounting for other variables that could influence suicidal risk.

## Discussion

SAD is a common mental health condition that has garnered increasing attention due to its impact on quality of life and mental health. Previous studies have highlighted a notable prevalence of this disorder, particularly among young people, with variations across age groups. In a recent meta-analysis, the prevalence of SAD was estimated to be 17% among young people, with lower rates among adolescents (8.4%) and children (4.7%).^17^ These results suggest a gradual increase in prevalence across different stages of development.

In our study, we observed a prevalence of 9.9% (95% CI: 8.3–11.8) among students over the age of 18, a rate lower than that reported by Salari et al.^17^ However, this rate is nearly identical to the one observed among students at Cumhuriyet University, in Turkey, which was 9.6%.^18^ However, available data shows significant variability depending on the context and the populations studied. For example, a meta-analysis involving 2878 participants from 7 studies estimated the overall prevalence of social anxiety disorder among students in Ethiopia to be 26.81% (95% CI: 22.31–31.30).^3^ Similarly, a study conducted in Australia reported a prevalence of 30%,^5^ which is notably higher than the rate observed in our sample. Furthermore, another study conducted among medical students in Sergipe, Brazil, revealed an even higher prevalence of 30.7%,^19^ a result consistent with data reported in Saudi Arabia among medical students (29.3%).^20^

These disparities in prevalence can be attributed to several factors, including methodological differences in the assessment of SAD, as well as academic, psychosocial and cultural contexts specific to each population studied, influencing the expression of social anxiety. For example, the high prevalence observed among medical students in Brazil and Saudi Arabia^19,20^ may be explained by their frequent exposure to stressful situations requiring social interactions, such as oral exams, clinical placements or interactions with patients and supervisors.

Moreover, methodological variations between studies can also play an important role. In our study, as well as in the one conducted in Turkey,^18^ the evaluation relied on semi-structured interviews, while other works, such as those conducted in Australia and Brazil,^5,19^ used a self-administered screening scale, which could overestimate the prevalence of the disorder. This is why our prevalence was similar to that of Brazil, while in Australia and Brazil the rates were higher, reaching 30%.

Additionally, cultural factors significantly influence the prevalence and expression of social phobia, particularly through the dimensions of individualism and collectivism. Research suggests that collectivist cultures, such as those in Korea, Japan, Saudi Arabia and Ethiopia, report higher levels of social anxiety symptoms compared with individualist cultures like Australia and the USA.^21^ In these collectivist societies, where group harmony is a core value, the fear of negative evaluation is more pronounced, thus reinforcing the avoidance of anxiety-inducing social situations.^22^ This cultural influence could partly explain the prevalence gaps observed between different studies and regions of the world.

### Social anxiety disorder and suicidal risk

Our results highlight a significant association between social anxiety and suicidal risk. Participants with SAD had significantly higher rates of suicidal ideation (27.9 *v*. 6.9%; *p* < 0.001), suicide attempts in the previous month (5.5 *v*. 0.9%; *p* < 0.001) and lifetime suicide attempts (11.6 *v*. 4.6%; *p* = 0.002). Furthermore, nearly one-third (28.7%) of individuals with SAD had a suicidal risk, a prevalence three times higher than that of participants without social phobia (9.4%; *p* < 0.001).

Even after adjustment in a multivariate analysis controlling for confounding variables such as depressive episodes and psychiatric history, SAD remained an independent risk factor for suicidal risk. These results reinforce the hypothesis that social anxiety itself, and not just depressive comorbidity, constitutes a vulnerability factor for suicidal thoughts and behaviours.

However, some studies highlight the importance of depressive comorbidity in this relationship. Indeed, social anxiety is frequently associated with depression, with more than 20% of individuals with SAD also suffering from a major depressive episode.^12,13^ For instance, Mathialagan et al observed that among adolescents with major depression comorbid anxiety disorders, including social anxiety, were not significantly associated with suicidal behaviours after adjusting for depression.^23^ However, our observations align with the existing literature that also shows a significant association between social anxiety and suicidal thoughts as well as suicidal risk. A meta-analysis by Leigh et al revealed a significant association between social anxiety and suicidal thoughts (*r* = 0.22) as well as with suicidal risk (*r* = 0.24).^10^ Similarly, another meta-analysis by Bentley et al identified social anxiety as a risk factor for suicidal thoughts and attempts.^9^ Cross-sectional studies have corroborated these findings, showing that social anxiety disorder is associated with an increased risk of suicidal behaviours.^24,25^ A prospective study also revealed a significantly higher number of suicide attempts among individuals suffering from both major depression and SAD, compared with those suffering only from depression.^26^ Furthermore, Gallagher et al observed that social anxiety symptoms prospectively predicted suicidal ideation after 18 months of follow-up, even after adjusting for depression.^27^

Among patients with bipolar disorder, the presence of social anxiety disorder also increases the risk of suicide attempts, independently of depressive episodes.^28,29^

Social anxiety seems to influence suicidality through interpersonal distress and social isolation. In adults, Brown et al demonstrated that the relationship between social anxiety and suicidal ideation was primarily mediated by relational difficulties, reinforcing the hypothesis that social avoidance and lack of support are key mechanisms of suicidal risk.^11^ These findings are consistent with those of Motillon-Toudic et al, who highlight that social isolation increases suicidal vulnerability, while adequate social support plays a protective role.^30^

### Strengths and limitations

Our study presents several strengths. To our knowledge, it is the first research conducted in Morocco to explore the association between social anxiety and suicidal risk, thus addressing a significant gap in the existing literature. It is based on a highly representative multicentre sample, with high statistical power that reinforces the validity of the findings. The use of face-to-face semi-structured interviews, a validated and widely recognised scale in international research and strict adherence to the ethical principles of the Declaration of Helsinki contribute to particularly relevant and reliable results.

However, the cross-sectional nature of the study constitutes a limitation. The use of self-reported data may introduce reporting bias, particularly concerning histories of substance use or psychiatric follow-up. In addition, we acknowledge that the version of the MINI used is based on DSM-IV criteria, which represents a methodological limitation in light of the current DSM-5 classification.

In conclusion, our results show that SAD is a major risk factor for suicidal behaviours. They emphasise the need for early identification and appropriate management of SAD among students in order to prevent suicidal risks. It is crucial to implement targeted prevention strategies within university settings to promote students’ mental health and reduce the risk of suicidal behaviours.

## Data Availability

The data that support the findings of this study are available on request from the corresponding author, S.E. The data are not publicly available due to their containing information that could compromise the privacy of research participants. The analytic code supporting the findings of this study is available upon reasonable request from the corresponding author, S.E.
